# Intratumoral Fibrosis in Facilitating Renal Cancer Aggressiveness: Underlying Mechanisms and Promising Targets

**DOI:** 10.3389/fcell.2021.651620

**Published:** 2021-03-11

**Authors:** Chao Hu, Yufeng Zhao, Xuanchuan Wang, Tongyu Zhu

**Affiliations:** ^1^Department of Urology, Zhongshan Hospital, Fudan University, Shanghai, China; ^2^Shanghai Key Laboratory of Organ Transplantation, Shanghai, China

**Keywords:** cancer-associated fibroblast, intratumoral fibrosis, metabolism, microRNA, renal cell carcinoma

## Abstract

Intratumoral fibrosis is a histologic manifestation of fibrotic tumor stroma. The interaction between cancer cells and fibrotic stroma is intricate and reciprocal, involving dysregulations from multiple biological processes. Different components of tumor stroma are implicated via distinct manners. In the kidney, intratumoral fibrosis is frequently observed in renal cell carcinoma (RCC). However, the underlying mechanisms remain largely unclear. In this review, we recapitulate evidence demonstrating how fibrotic stroma interacts with cancer cells and mechanisms shared between RCC tumorigenesis and renal fibrogenesis, providing promising targets for future studies.

## Introduction

Renal fibrosis is the common outcome of different chronic kidney diseases (CKDs), characterized by excessive accumulation of extracellular matrix (ECM) and disrupted renal microarchitecture (Hewitson, 2009). Formation of fibrosis involves numbers of cell subtypes, including epithelial, endothelial, and inflammatory cells with a purpose to trigger fibrosis and fibroblasts, pericytes that execute fibrosis (Lovisa et al., 2016). The intricate cross-talk between these cells has been brought to understanding but still remains largely controversial.

Renal cell carcinoma (RCC) is one of the most common malignancies. It accounts for 85% of kidney neoplasms (Cancer.Net, 2020), the global incidence of which was estimated to be 403,000 in 2018 worldwide (Bray et al., 2018). It is classified into mainly three subtypes, namely, clear cell RCC (ccRCC), papillary RCC (PRCC), and chromophobe RCC. The identification of von Hippel–Lindau (*VHL*) in ccRCC has furthered our understanding of the underlying mechanisms of RCC formation. Tumor suppressor VHL serves as a substrate recognition subunit of a ubiquitin ligase targeting hypoxia-inducible factor (*HIF*). Inactivation of *VHL* results in abnormal stabilization of HIF pathway, favoring atypical cell growth through promoting cell survival under hypoxia condition (Kaelin, 2008).

As a persistent tissue injury, cancer cells initiate a chronic wound healing response in tumors, namely, intratumoral fibrosis (ITF). ITF is the result of aberrant accumulation of collagen matrix produced by cancer-associated fibroblasts (CAFs) (Liu et al., 2019b). As a highly vascularized tumor, RCC is frequently found with ITF. Joung et al. (2018) reported that among 204 ccRCC cases, 167 (81.7%) showed ITF. Although the correlation between prognosis of ccRCC and ITF is not significant, ITF proves to be related to other poor prognostic factors in ccRCC including Fuhrman nuclear grade, intratumor necrosis, and lymphovascular invasion. It is safe to acknowledge the contribution of tumor cell microenvironment in tumorigenesis. A wide range of studies have been conducted to elucidate the underlying interactions between fibrosis and cancer. The microenvironment surrounding tumor cells serves as both powerful tumor suppressor and tumor promoter (Sternlicht et al., 1999). Fibroblasts, the dominant component of tumor stroma, were proved to induce oncogenic potential of adjacent epithelia (Bhowmick et al., 2004) and associated with early and advanced stages of tumor progression (Rønnov-Jessen et al., 1996; Giussani et al., 2015). Formation of mature tumor ECM is marked by high density of fibrillar collagens, especially type I collagen, and capable of resisting degradation and repetitive mechanical stress (Yamauchi et al., 2018). Cancer cells trigger the formation of tumor stroma and stiffening stroma benefits tumor growth in return, suggesting that the dynamics between stroma and cancer cells is mutual. Evidence in different organs sustains that instead of merely preceding or tailing cancer formation, fibrosis participates in the cancer formation and metastasis (Neesse et al., 2015; Saito et al., 2018; Tzouvelekis et al., 2019). However, the evidence describing the correlation between cancer and fibrotic stroma both clinically and mechanically in kidney is limited.

In this review, we introduce how fibrotic stroma interacts with tumor cells in different organs: (1) the interplay between fibrotic stroma and cancer cells via metabolic manners; (2) how signaling mediates features of fibronectin (FN) and enzymes regulating collagen exert a protumor effect; (3) robust reciprocal communications between cancer cells and CAFs mediated by secretory molecules; (4) demonstration of the pro-inflammatory feature of CAFs and the controversial involvement of ECM in tumor immunity. Next, we focus on demonstrating the potential role of different signaling pathways including mammalian target of rapamycin (mTOR), Wnt, and Notch and molecules including non-coding RNA (ncRNA), fumarate hydratase (FH), and other molecules, promoting both renal fibrosis and RCC, which hopefully may provide valid insights for future studies regarding the correlation between these two pathogeneses.

## The Relationship Between Tumor and Stroma

### Metabolic Interaction Between Cancer Cells and Stroma

Fibrotic stroma drives metabolic shifts in cancer cells, fostering multiple malignant features. After being activated, CAFs also shift to aerobic glycolysis (Vander Heiden et al., 2009). CAFs have been shown to promote glycolysis in ovarian cancer cells by inducing phosphorylation and activation of phosphoglucomutase 1, facilitating proliferation and metastasis (Curtis et al., 2019). Aspartate generated by CAFs is shown to promote tumor proliferation. In return, glutamate secreted by tumor cells contributes to maintaining redox homeostasis of CAFs through glutathione pathway (Bertero et al., 2019). Involvement of lactate and pyruvate in promoting the cell growth is also identified in different cancer cell types (Sanford-Crane et al., 2019). In addition to aberrant secretion from CAFs, alterations in ECM exert a certain influence on cancer cell metabolism. Increased collagen density in ECM was shown to be associated with decreased oxygen consumption and glucose metabolism in breast cancer cells (Morris et al., 2016). Degradation of hyaluronan promoted glucose uptake in several cancer cell lines. Induction of glycolysis by hyaluronidase accelerated cell migration (Sullivan et al., 2018).

### The Protumor Effect of Fibronectin and Collagen

The components of tumor stroma contribute to various tumor hallmarks. Tumor-associated stroma rich in FN and type I collagen was proved to be associated with enhanced cancer progression (Li et al., 2003). As the adhesion protein, FN provides the basic scaffold for nascent collagen deposition by fibroblasts, which is crucial to regulate cell proliferation and migration (Sottile and Hocking, 2002). FN plays a significant role in directing signals, by binding to a wide range of growth factors including transforming growth factor-β (TGF-β) superfamily, fibroblast growth factor (FGF) family, insulin-like growth factor binding protein-5 (IGFBP-5), and IGFBP-3 via FN III12–14, a highly promiscuous GF binding domain (Martino and Hubbell, 2010). FN-rich ECM drives desmoplastic differentiation of normal fibroblasts (Amatangelo et al., 2005). In RCC, FN was shown to promote cell growth and migration in part via Src and TGF-β1 signaling *in vitro*, the mechanism of which was not clearly demonstrated (Ou et al., 2019).

As the most abundant ECM scaffolding protein in the stroma, collagen is significantly associated with the tensile strength (Kolácná et al., 2007). Type I collagen protected against tumor invasion, while increased collagen I expression was related to elevated incidence of metastasis (Ramaswamy et al., 2003). Lysyl oxidase (LOX) and LOX-like (LOXL) family members initiate collagen cross-linking by catalyzing the oxidative deamination of Lys and Hyl residues and are found elevated in different tumors (Erler et al., 2009). Levental et al. (2009) proved that LOX-mediated collagen cross-linking, final step of collagen biosynthesis, stiffened the matrix, thereby promoting focal adhesions and tumor progression. Cox et al. (2013) showed that cross-linking of collagen I enhanced metastatic growth and that LOX-mediated collagen cross-linking increased tumor cell proliferation and metastasis. In RCC, studies showed that procollagen-lysine, 2-oxoglutarate 5-dioxygenases1/2/3 (PLOD1/2/3), and LOXL2, both collagen-modifying enzymes, were related to high pathological grades; however, the underlying mechanisms are vaguely depicted (Hase et al., 2014; Xu et al., 2019). Lysyl hydroxylase 2 (LH2), which is responsible for the overhydroxylation of the collagen telopeptides (van der Slot et al., 2004), shifted the tumor stroma toward high-Hylald-derived collagen cross-links, low-Lysald-derived collagen cross-link state, increasing tumor stiffness, and enhanced tumor cell invasion and metastasis (Chen et al., 2015). This evidence suggests both that the quality and the quantity of collagen are related to tumor progression via different mechanisms.

### Cancer-Associated Fibroblast and Cancer Cells

Among all the stromal cells, CAFs share most the intricate relationship with cancer cells. Under the unabated influence of a large array of stimuli, e.g., growth factors, cytokines, and chemokines, normal fibroblasts get activated into CAFs irreversibly. CAFs display promoted secretory phenotypes, ECM remodeling ability, and immunomodulatory functions, which regulate different cancer traits (Kalluri, 2016).

Stromal cell-derived factor 1 (SDF-1) secreted by CAFs was found to accelerate tumor growth directly and promote angiogenesis via recruiting endothelial progenitor cells (Orimo et al., 2005). In RCC, under hypoxic conditions, accumulation of HIF-1α upregulated chemokine receptor 4 (CXCR4), the receptor of SDF-1, leading to elevated metastatic ability (Pan et al., 2006). This evidence suggested that SDF-1/CXCR4 biological axis regulated organ-specific metastasis of RCC. As ECM-degrading proteases, matrix metalloproteinase (MMP)-1 and 3 produced by the CAFs contribute to tumor invasiveness (Lochter et al., 1997; Boire et al., 2005). A similar correlation was reported in RCC. Paracrine platelet-derived growth factor-CC (PDGF-CC) signaling pathway was reported to control breast cancer basal-like subtype (Roswall et al., 2018). The evidence of CAF secretion enhancing tumorigenesis is numerous and comprehensive. In breast cancer, cancer-derived osteopontin and WNT7A activated mesenchymal stem cells into CAFs and enhanced invasive features of CAFs, respectively, in a TGF-β-dependent manner (Weber et al., 2015; Avgustinova et al., 2016). ccRCC cells induced CAF-derived periostin expression, and elevated periostin promoted tumor cell itself and CAF proliferation, in return (Bakhtyar et al., 2013). Taken together, these evidences indicate a robust reciprocal relationship between cancer cells and CAFs. Particularly interesting is the physical force that CAFs exert on cancer cells promoting cancer invasion, via E-/N-cadherin adhesion (Labernadie et al., 2017).

Given the mounting publications delineating protumor effects of CAFs, it is reasonable to assume that increased fibrosis is positively associated with poor prognosis. However, signs of cancer cells progression being impeded by the tumor stroma have also been observed. In non-small cell lung carcinoma (NSCLC), a correlation of increased desmoplasia with longer survival was observed (Paulsson and Micke, 2014). Moreover, in pancreatic ductal adenocarcinoma cells, deletion of sonic hedgehog (*SHH*), an overexpressed soluble ligand driving formation of a fibroblast-rich desmoplastic stroma, results in more malignant features. The tumor-suppressing effect could be partially due to the unique capability of Hedgehog-driven stroma to restrain tumor angiogenesis (Rhim et al., 2014). Slit2 and Asporin, both secreted by stromal fibroblasts, were identified as tumor suppressor in breast cancer. Slit2, a ligand of Robo1 receptor, was found to restrain tumorigenesis via blocking PI3K/AKT/β-catenin pathway (Chang et al., 2012). High expression of Asporin, an inhibitor of TGF-β1, was significantly associated with less aggressive tumors (Maris et al., 2015). However, the exact subtype of stromal fibroblasts responsible for expressing Slit2 and Asporin remains to be determined. More studies are required to demonstrate the tangled functions of CAFs.

### Fibrotic Stroma and Cancer Immunity

As another essential component of tumor stroma, immune cells receive heated attention following the success of novel immunotherapies targeting adaptive immune system. Chemokine ligand 12 (CXCL12) solely produced by CAFs was shown to negatively regulate T-cell accumulation. By targeting it, a promising synergistic effect with anti-PD-L1 immunotherapy was observed in pancreatic cancer (Feig et al., 2013). On the other hand, the innate immune system is of great significance as well, given its dynamic reciprocity between fibrosis and inflammation (Alexander and Cukierman, 2016).

CAFs regulate hallmark features of tumor by mediating tumor-promoting inflammation. A large array of cytokines and chemokines are related to CAFs and exert pro-inflammatory effects (Servais and Erez, 2013; Acerbi et al., 2015). Pro-inflammatory gene signature has been identified in CAFs in different organs, and the underlying mechanisms are becoming understood. CAFs were shown to promote tumor growth and macrophage recruitment with participation of nuclear factor-kappaB (NF-κB) signaling pathway (Erez et al., 2010). More evidence suggests that CAFs induce Th2 and Th17 inflammation response in a thymic stromal lymphopoietin (TSLP)-dependent manner and TLR, nucleotide oligomerization binding domain 2 signaling, respectively (Su et al., 2010; De Monte et al., 2011). In contrast to various cross-talks between immune cells and CAFs, fibrotic ECM serves as a barrier against immune cells. Matrix areas packed with aligned fiber and collagen hindered migration of T cells, blocking them from approaching cancer cells (Salmon et al., 2012; Hartmann et al., 2014; Chen et al., 2018). However, under the assistance of a novel computational imaging technology, Carstens et al. (2017) discovered no positive correlation between T-cell accumulation and collagen-I, α-SMA fibroblasts. Further investigation is required to determine the specific contribution of each component of ECM and to explore corresponding therapeutic treatments.

Cancer cells trigger the alterations in stroma. A reciprocal relationship is identified in all four sections, especially a notably beneficial interaction between CAFs and cancer cells. To various degrees, most components of fibrotic stroma including infiltrating lymphocytes are implicated. Secretory molecules play a vital part in the communications between fibrotic stroma and cancer cells owing to their capability of recruiting and activating the target cells. Tumor immunity and metabolism both contribute greatly to RCC tumorigenesis. Evidence revealed how metabolic alterations in RCC affected tumor immune microenvironment (Xiao and Meierhofer, 2019). However, no effort has been made to determine whether fibrotic stroma re-shapes the tumor immunity in RCC as well. Crossing these two fields yields a promising direction for future exploration.

## Mechanisms Shared Between Renal Cell Carcinoma Tumorigenesis and Renal Fibrogenesis

### Wnt Signaling

Initiated by Wnt ligands binding to the extracellular domain of frizzled (Fzd) receptor and co-receptors, low-density lipoprotein receptor-related proteins 5 and 6 (LRP5 and LRP6), the canonical Wnt signaling depends on the intracellular molecular β-catenin to exert its influence on multiple biologic processes (Clevers and Nusse, 2012). Wnt ligands embrace 19 different members; and 16 of them, except Wnt3a, Wnt8a, and Wnt10b, are upregulated in the unilateral ureteral obstruction (UUO) model (He et al., 2009). Zhou et al. (2017) reported that blocking the WNT secretion in renal tubular cells reduced β-catenin activation and inhibited myofibroblast activation *in vivo*, whereas blocking in fibroblasts showed little effect, suggesting that Wnt/β-catenin signaling displays its functions in the tubular epithelium in the renal fibrotic diseases. *WNT1* has been reported to be related to both RCC and renal fibrosis. Not only high WNT1 was associated with more advanced stage, increased size, and overall survival, but it also promoted renal fibroblast proliferation *in vitro* (Kruck et al., 2013; Maarouf et al., 2016). However, few research on WNT1 has been conducted to explore the interaction between the two major diseases so far. Moreover, WNT2, WNT3A, and WNT4 were shown to induce fibroblast proliferation and myofibroblast differentiation *in vitro*, respectively (DiRocco et al., 2013; Zhou et al., 2017). WNT10A expression induced RCC cell proliferation and aggressiveness, while WNT7A displayed tumor suppression function *in vitro* (Hsu et al., 2012; Kondratov et al., 2012). Abnormal accumulation of β-catenin was related to both renal fibrogenesis and RCC carcinogenesis (Kruck et al., 2013; Maarouf et al., 2015). On the other hand, the expression of *Fzd7* and mRNA expression of *Fzd5* and *8* were shown to be upregulated in RCC and contributed to cell proliferation (Janssens et al., 2004; Xu et al., 2016), while no *Fzd* genes were repressed after obstructive injury, suggesting an underlying correlation to explore (He et al., 2009). Extensive studies have determined the functions of different components of Wnt signaling in renal fibrosis and RCC, whereas the interaction between these two fields has been rarely explained.

### Mammalian Target of Rapamycin Signaling

The mTOR is a component of two distinct complexes, mTOR complex 1 (mTORC1) and mTORC2. As an evolutionarily conserved serine–threonine kinase, mTOR regulates cell growth, proliferation, autophagy, and metabolism (Ma et al., 2018). AMP-activated protein kinase (AMPK) and PI3K-AKT-dependent pathways converge on tuberous sclerosis complex (TSC), which subsequently activates mTORC1 by releasing Rheb, a Ras family GTPase. The well-described downstream factors of mTORC1 include p70S6K and 4EBP, which favor cell growth and proliferation via enhancing proteins and nucleotide synthesis (Fantus et al., 2016). Mechanically, mTORC1 is better characterized in both kidney malignancy and fibrosis. Chen et al. (2012) determined the interstitial macrophages and myofibroblasts as the main cell subtypes with persistent activation of mTORC1 signaling. Decreased levels of profibrotic cytokines, including TGF-β1, VEGF, glomerular connective tissue growth factor, and monocyte chemoattractant protein-1, were observed in models treated with rapamycin *in vivo* (Lloberas et al., 2006; Yang et al., 2007; Liu et al., 2014). Rapamycin was proved to reduce tubulointerstitial fibrosis in the UUO model and block TGF-β1-induced loss of E-cadherin expression, suggesting that mTOR signaling also contributed to the transdifferentiation from tubular epithelial cells to α-SMA-positive myofibroblasts (Wu et al., 2006). In addition to mTORC1, the engagement of mTORC2 in renal fibrogenesis was also recognized. Li et al. (2015) reported that Rictor/mTORC2 signaling induced TGF-β1-promoted fibroblast activation independent of mTORC1 signaling, indicating that both mTORC signaling was involved in the fibroblast response to TGF-β1.

As an intermediate regulator, a wide range of molecules contribute to RCC different malignant phenotypes via mTOR signaling pathway, including pyruvate kinase M2 (PKM2) (Dey et al., 2019), enoyl-CoA hydratase short-chain 1 (ECHS1) (Qu et al., 2020), nucleobindin-2 (NUCB-2) (Tao et al., 2020), miR-100 (Liu et al., 2020b), and maternal and embryonic leucine zipper kinase (MELK) (Zhang et al., 2019). Mutations in upstream factors of mTOR signaling pathway were also involved. Phosphatase and tensin homolog deleted on chromosome 10 (PTEN) mutation correlated with high-grade, advanced ccRCCs with enhanced ability of invasion (Kondo et al., 2001). In addition to the well-established TSC-mTOR signaling, Brugarolas et al. (2003) revealed an mTOR-independent pathway, possibly associated with chromatin remodeling. Intriguingly, epithelial–mesenchymal transition (EMT) was induced though mTOR pathway in two diseases (Wu et al., 2006; Tao et al., 2020).

Currently, use of mTOR inhibitor is mainly restricted to patients with advanced RCC and refractory to anti-VEGF therapy. Temsirolimus and everolimus both targeting mTORC1 were put into clinical use (Hudes et al., 2007; Motzer et al., 2008). In order to avoid activation of phosphatidylinositol 3-kinase (PI3K)/AKT initiated by sole inhibition of mTORC1, novel mTOR ATP-competitive blocker AZD-2014 targeting mTORC1/2 was developed and showed superior potency to restrain RCC cell growth both *in vivo* and *in vitro* as compared with mTORC1 inhibitor (Zheng et al., 2015). Interestingly, AZD-2014 activated cancer cells autophagy, which could prolong cancer cells survival. Co-administration of autophagy inhibitor 3-MA enhanced AZD-2014 growth arrest effect. However, in the randomized Phase II study, AZD-2014 failed to surpass everolimus in progression-free survival and overall survival in patients with VEGF-refractory metastatic ccRCC (Powles et al., 2016). Autophagy was also found to be activated in CAFs and foster tumor progression via modulating secretory factors including IL-6 and IL-8 in head and neck cancer (New et al., 2017). Focusing on the combined therapy of deactivation of autophagy in both cancer cells and tumor stroma and developing novel mTORC1/2 dual inhibitor could forward mTOR inhibitors to overcome resistance and display better efficacy in clinical trials.

### Non-coding RNA

NcRNAs is divided into two classes mainly by their length: small (<200 nucleotides) and long (>200 nucleotides) ncRNAs. MicroRNAs (miRNAs) are included in the small ncRNAs, along with small interfering RNAs and small nucleolar RNAs (Mattick and Makunin, 2006). MiRNAs are short ncRNAs that modulate various physiological and pathological processes by negatively regulating the expression of their target genes via blockade of protein translation or by inducing mRNA degradation (Ambros, 2004). The studies of miRNAs profiling shed some light on the role of miRNAs in RCC tumorigenesis, while the underlying mechanism has not been well-demonstrated. A fraction of studies merely predicted the target genes of dysregulated miRNA using different analysis approaches without verifying it experimentally. Some of these genes are related to RCC tumorigenesis, remaining to be promising directions (Table 1). On the other hand, efforts have been made to delineate how miRNAs contribute to fibrosis, mostly in diabetic nephropathy (DN). Multiple studies we detected overlap on the E-box repressor such as δEF1, Smad-interacting protein 1 (SIP1), zinc finger E-box binding homeobox 1 (ZEB1), and ZEB2, indicating its significant role as a mediator in TGF-induced fibrosis (Table 2).

**TABLE 1:** Studies of miRNA in renal cell carcinoma.

**MicroRNA**	**Mechanisms**	**Sources**	**References**
MiR-122, 155, 21, and 210	Overexpression (predicted targets including HIF-1α, VEGF receptor 2, mTOR, etc.)	PS	Juan et al., 2010; White et al., 2011a
MiR-200c, 335, 199, and 218	Downregulated (predicted targets including AKT, RAS, Rheb, etc.)	PS	Chow et al., 2010; White et al., 2011a
MiR-141 and 200c	Inhibit E-cadherin expression via a ZHFX1B-mediated transcriptional repression	PS	Nakada et al., 2008
MiR-215	Negatively regulate cellular migration and invasion	*In vitro*	White et al., 2011b
MiR-192, 194, and 215	Suppress tumor progression convergently	*In vitro*	Khella et al., 2013a
MiR-377	Reduce cell proliferation, migration, and invasion by targeting ETS1	*In vitro*	Wang et al., 2015
MiR-29s	induce cell migration and invasion via miR-29s–LOXL2 axis	*In vitro*	Nishikawa et al., 2015
MiR-93	Inhibits apoptosis and promotes proliferation, invasion, and migration via TGF-β/Smad signaling	*In vitro*	Liu et al., 2017

*PS, patient specimens; miRNA, microRNAs; mTOR, mammalian target of rapamycin; EMT, epithelial–mesenchymal transition; TGF, transforming growth factor.*

**TABLE 2:** Studies of miRNAs in renal fibrosis.

**MicroRNA**	**Mechanisms**	**Sources**	**References**
MiR-200s	Protect tubular epithelial cells from mesenchymal transition via A. targeting ZEB1 and ZEB2 B. downregulated in a TGF-β1/Smad signaling-dependent manner	*In vitro*	Xiong et al., 2012
MiR-192	Controls TGF-β-induced fibrosis via mediating E-box repressor A. SIP1 and δEF1 B. ZEB1 and ZEB2 C. ZEB2 (with miR-215)	*In vivo* and *in vitro*; *In vivo*; *In vitro*	Kato et al., 2007; Krupa et al., 2010; Wang et al., 2010
MiR-377	Increases fibronectin protein production	*In vivo* and *in vitro*	Wang et al., 2008
MiR-29a MiR-29b	A. Negatively regulates collagen IV by directly binding to its 3’-UTR B. A downstream inhibitor of TGF-β /Smad3-mediated fibrosis C. Regulates Ang II-induced EMT via targeting PI3K/AKT signaling pathway	*In vitro*; *In vivo* and *in vitro; In vitro*	Qin et al., 2011; Wang et al., 2012; Hu et al., 2018
MiR-93	Abrogate VEGF downstream targets, collagen IV, and fibronectin	*In vivo* and *in vitro*	Long et al., 2010

*miRNA, microRNAs; TGF, transforming growth factor; EMT, epithelial–mesenchymal transition.*

Nakada et al. (2008) reported that downregulation of miR-141 and miR-200c in ccRCC suppressed CDH1/E-cadherin transcription via upregulation of ZEB2, also known as ZFHX1B. MiR-200a and miR-141 were shown to abrogate EMT of tubular epithelial cells by targeting ZEB1 and ZEB2, revealing an anti-fibrotic effect of the miR-200 family (Jung et al., 2009). This evidence demonstrated that miR-200 family was involved in mediating the transcriptional repressor of E-cadherin and induction of EMT, leading to RCC and renal fibrosis separately. Additionally, several studies identified engagement of multiple miRNAs including miR-382 (Kriegel et al., 2010), miR-23a (Xu et al., 2018), and miR-133b and 199b (Sun et al., 2018) in renal fibrogenesis or tumorigenesis via EMT, suggesting EMT as a promising target to bridge two diseases.

Both classes are well-established to exert a certain influence on various biological processes, and the interactions among them are coming into the view (Yoon et al., 2014). Long ncRNAs (lncRNAs) regulated miRNA function by acting as miRNA sponges and inhibiting their binding to target mRNAs (Paraskevopoulou and Hatzigeorgiou, 2016). Fibrogenic effects of lncRNAs were observed in several CKD models. Liu et al. (2020a) showed that metastasis-associated lung adenocarcinoma transcript 1 (MALAT1)/miR-145/focal adhesion kinase (FAK) pathway was implicated in TGF-β1-induced renal fibrosis in obstructive nephropathy. MALAT1 regulated high glucose (HG)-induced EMT and fibrosis by functioning as a sponge RNA for miR-145, resulting in derepressing the expression of target gene ZEB2 (Liu et al., 2019a). Multiple publications revealed how MALAT1 contributed to RCC tumorigenesis (Xiao et al., 2015; Zhang et al., 2015). Hirata et al. (2015) reported that MALAT1 promoted RCC progression via Ezh2, the potential binding protein of MALAT1, and interacting with miR-205, which led to blockage of EMT via E-cadherin recovery and β-catenin downregulation. Additionally, various lncRNAs have been confirmed to facilitate processes such as cell migration, metastasis, invasion, proliferation, and apoptosis verified in different cell lines (Moghaddas Sani et al., 2018).

New targets and functions of miRNAs are being determined at a tremendous rate; however, our understanding of miRNAs fails to go further correspondingly. The overlap of target gene prediction using different algorithms is far from satisfactory, and subsequent experimental validations are inadequate. Moreover, one target gene could be controlled by multiple miRNAs, and vice versa (Khella et al., 2013b). Studies focusing on convergent and divergent effects of certain miRNAs would be more eloquent to elucidate how such network regulates its target gene. On the grounds that lncRNAs display its function partly through regulating miRNAs, it is paramount to launch studies delineating the network interactions between the two different classes of ncRNAs, hopefully reaching a more comprehensive understanding.

### Notch Signaling

The Notch signaling pathway is an evolutionarily conserved signaling pathway, composed of four Notch receptors (Notch 1–4) and five ligands [delta-like ligand (DLL)-1, DLL-3, DLL-4, Jagged-1, and Jagged-2] (Artavanis-Tsakonas et al., 1999). Its role in renal malignancy and fibrosis was demonstrated separately. Huang et al. (2018) recognized that *Jagged1* and *Notch2* contributed to kidney fibrosis development by regulating mitochondrial transcription factor A (*Tfam*) expression and metabolic reprogramming. Notch-induced kidney fibrosis was related to metabolic dysregulations and could be restored by peroxisomal proliferator-g coactivator-1a (PGC-1a). The downstream target of Notch1 signaling, Hes1, was capable of regulating PGC-1a directly (Han et al., 2017). Both publications revealed aberrant metabolism disturbed by activated Notch signaling, resulting in a profibrotic feature.

High-level expression of Notch signaling positively correlates with tumor size, nuclear grade, and TNM stage and risk of metastasis in T1 stage ccRCC (Wu et al., 2011; Ai et al., 2012). JAGGED1 and 2 were confirmed to be associated with loss of CpG methylation of H3K4me1-associated enhancer regions and gene amplification, respectively, indicating that the activation of Notch signaling pathway could result from both genetic and epigenetic alterations (Bhagat et al., 2017). Activated Notch signaling was identified in renal cancer stem cells by both transcriptional profiling and single-cell sequencing (Fendler et al., 2020). Xiao et al. (2017) showed that overexpression of Notch1 exerted an upregulatory impact on chemotaxis of RCC cancer stem cells via SDF-1/CXCR4 axis.

### Fumarate Hydratase

In terms of FH, extensive researches have been carried out to demonstrate how such mutations of metabolic enzymes engage in hereditary cancer syndromes. FH inactivation was proved to predispose individuals to hereditary leiomyomatosis and renal cell cancer (HLRCC) (Kim and Kaelin, 2006). In addition to Krebs cycle, further studies spotted significant changes in the urea cycle and determined cytosolic metabolic pathways in FH-associated oncogenesis (Adam et al., 2013). Both *in vivo* and *in vitro* evidence supported that accumulation of fumarate caused stabilization of HIF-1α (Pollard et al., 2005). However, based on a much more thorough study, a distinct mechanism of Fh1-dependent, the murine homolog of FH, cyst formation was proposed. Adam et al. (2011) provided solid evidence asserting the absence of Hif/Phd pathway and introducing nuclear factor-like 2 (NRF2) dysregulation as an oncogenic pathway involved in FH-associated disease. Interestingly, FH inactivation also engages in renal fibrosis. Reduction of FH caused the accumulation of fumarate, leading to fibrosis in DN in Goto–Kakizaki (GK) rats. Increased levels of HIF-1α and TGF-β1 were detected, suggesting candidate mechanisms accounting for such fibrosis (Miura et al., 2019). Although these studies on FH inactivation leading to renal carcinoma and fibrosis were conducted separately, it still provided insights to bridge our understanding of two major diseases.

### Other Molecules

Apart from the major signaling pathways and molecules that we mentioned above, several scattered individual studies also come into our view. YAP/TAZ is associated with the mechanical traits of the cell microenvironment, while not as well-described as the pathways we mentioned before. We detected two proteins regarding ECM remodeling, Basigin and MXRA5, suggesting a more comprehensive engagement of ECM in RCC tumorigenesis. High mobility group box 1 (HMGB1) is a nuclear protein that acts as a co-factor for gene transcription. As the major NADPH isoform in kidney, Nox4 contributes to redox processes by mainly producing H_2_O_2_. These publications receive less attention but still broaden our view and provide interesting insights to better demonstrate how RCC and fibrosis may interact with each other (Table 3).

**TABLE 3:** Molecules involved in renal cell carcinoma and fibrosis.

**Molecules**	**Mechanism (fibrosis/cancer)**	**Anti/pro**	**References**
HMGB1	A. TGF-β-mediated NLRP3-HMGB1 activation leads to tubulointerstitial fibrosis B. Facilitating ccRCC tumorigenesis via ERK1/2 activation, partially mediated by RAGE	Pro	Lin et al., 2012; Zhang et al., 2020
Basigin/CD147	A. Upregulating MMPs, TGF-β B. Upregulating proliferation and invasive potential by promoting VEGF and bFGF expression	Pro	Kato et al., 2011; Sato et al., 2013
MXRA5	A. Reducing the expression of gene encoding fibronectin and type IV collagen B. A direct relationship between VHL and MXRA5 transcriptional expression (underlying mechanism undetermined)	Anti	Poveda et al., 2017
Nox4	A. Involved in TGF-β1-induced EMT, mediated by RhoA/Rho kinase B. Promoting the expression and nuclear accumulation of HIF-2α	Pro	Gregg et al., 2014; Manickam et al., 2014
YAP/TAZ	A. Enhancing TGF-β-induced EMT and β-catenin expression B. Upregulating CYR61 and c-Myc	Pro	Schütte et al., 2014; Seo et al., 2016

*HMGB1, high mobility group box 1; TGF, transforming growth factor; ccRCC, clear cell renal cell carcinoma; MMP, matrix metalloproteinase; VHL, von Hippel–Lindau; EMT, epithelial–mesenchymal transition.*

All these signaling pathways and molecules are related to renal fibrogenesis and tumorigenesis, some components of which are directly involved in both pathologies. Future experiments focusing on these directions may be of importance to unveil how fibrotic stroma facilitates RCC aggressiveness. EMT is a canonical process that shifts the cancer cells into a mesenchymal phenotype, hence being a driver of the metastasis. TGF-β1 secreted by CAFs led to EMT of urinary bladder cancer cells through lncRNA-ZEB2NAT (Zhuang et al., 2015). This study revealed that tumor stroma could prompt cancer development via inducing EMT of cancer cells. As we demonstrated above, EMT and its secretory mediator TGF-β1 have been identified in two renal diseases repeatedly; however, in RCC, there has been no study conducted to determine whether EMT caused by tumor stroma is capable of facilitating RCC tumorigenesis. It remains to be a promising direction to explore ncRNA and different signaling pathways as demonstrated, which may further our understanding by elaborating the underlying mechanisms.

## Conclusion

**FIGURE 1:**
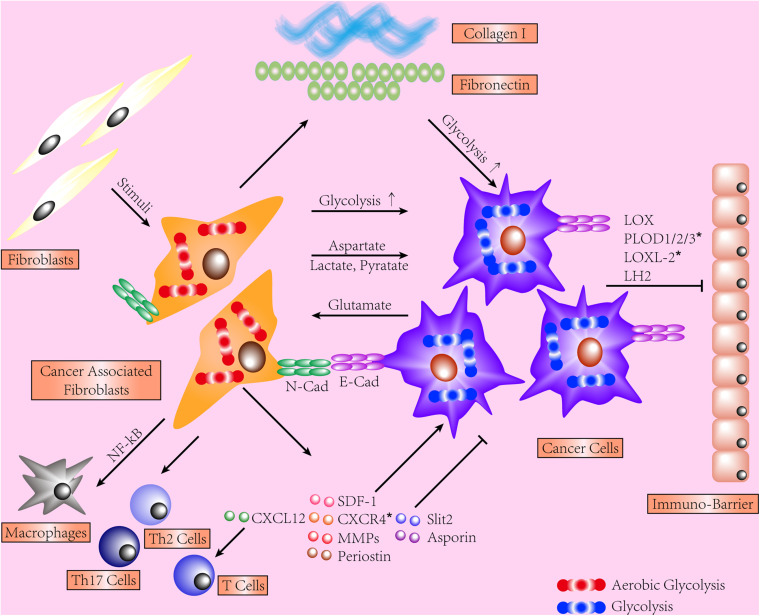
This schematic shows the interactions between stroma and cancer cells. Cancer-associated fibroblasts (CAFs) display an enhanced secretory phenotype, regulating immune cells and cancer cells and producing excessive extracellular matrix (ECM). Both CAFs and cancer cells shift to glycolysis and share a dynamic exchange of metabolites. The force transmission is mediated by E-cadherin/N-cadherin. Fibrotic ECM induces CAF activation, facilitates tumor invasion, and hinders T-cell migration. Type I collagen exerts a quantity-dependent pro- or anti-tumor effect on cancer cells. *Represents the data collected from renal cell carcinoma (RCC) models.

The contribution of tumor stroma to cancer cell is widely acknowledged. We provide evidence in different organs depicting how reciprocal interactions between cancer cells and fibrotic stroma function, few of which are regarding RCC. On the grounds that ITF was shown to correlate with several indicators of poor prognosis of ccRCC, it is logical to broaden our view of RCC by investigating the contribution of fibrotic stroma and delineating concerned mechanisms. We show that such reciprocal interactions are joint efforts from different dysregulations, including various components of excessive ECM, aberrant metabolisms, activation of CAFs, and tumor immunity (shown in Figure 1). Next, we recapitulate mechanisms shared between RCC progression, metastases, and formation of renal fibrosis. mTOR, Notch, Wnt signaling pathways, and ncRNA widely participate in RCC tumorigenesis and renal fibrogenesis via different manners. Additionally, secretory molecules and process of EMT are widely implicated and may be promising targets. The majority of publications we detected regarding the interactions between fibrotic stroma and cancer cells are based on experiments conducted in the lung, breast, pancreas, etc., suggesting an absence of deserved attention on the kidney. Hopefully, the evidence we collect may provide promising targets for future experiments.

## Author Contributions

CH and TZ conceived and designed the study and coordinated this work. CH, YZ, and XW collected, reviewed the literature, and composed the draft. CH, YZ, XW, and TZ organized the manuscript. All authors helped with data interpretation and manuscript editing.

## Conflict of Interest

The authors declare that the research was conducted in the absence of any commercial or financial relationships that could be construed as a potential conflict of interest.
